# Relationship between Lesion Parameters after Radiofrequency Catheter Ablation in Striated Muscles and Parenchymal Tissue

**DOI:** 10.3390/medicina60071089

**Published:** 2024-07-03

**Authors:** Aldona Gružienė, Julius Liobikas, Artūrs Paparde, Sigita Kerzienė, Jovita Gružaitė, Darijus Skaudickas, Povilas Lenčiauskas, Kristaps Circenis, Gintautas Vaitiekaitis

**Affiliations:** 1 Medical Academy, Lithuanian University of Health Sciences, LT-50161 Kaunas, Lithuania; arturs.paparde@lsmu.lt (A.P.); sigita.kerziene@lsmu.lt (S.K.); jovita.gruzaite@lsmu.lt (J.G.); darijusskaudickas@gmail.com (D.S.); povilas.lenciauskas@gmail.com (P.L.); vaitiekaitis.gintas@gmail.com (G.V.); 2 Department of Nursing and Midwifery, Rīga Stradiņš University, LV-1007 Riga, Latvia; kristaps.circenis@rsu.lv

**Keywords:** radiofrequency catheter ablation, electrode cooling, tissue damage, quotient, parenchymal tissue, striated muscle

## Abstract

*Background and Objectives*: Radiofrequency catheter ablation (RFCA) is a highly successful intervention. By comparing the lesion changes in prostate parenchymal and striated muscle tissues after RFCA with and without cooling, it was possible to assess the correlation between the shape regularity, area, and perimeter of the thermal lesion, and to predict the geometric shape changes of the lesions. *Materials and Methods*: A standard prostate and striated muscle RFCA procedure was performed on 13 non-purebred dogs in two sessions: no cooling and cooling with 0.1% NaCl solution. Microtome-cut 2–3 µm sections of tissue samples were stained with haematoxylin and eosin and further examined. The quotient formula was employed to evaluate the geometric shape of the damage zones at the ablation site. *Results*: The extent of injury following RFCA in striated muscle tissue was comparable to that in prostate parenchymal tissue. Regression analysis indicated a strong and positive relationship between area and perimeter in all experimental groups. In the experimental groups of parenchymal tissues with and without cooling, an increase in the area or perimeter of the damage zone corresponded to an increase in the quotient value. A similar tendency was observed in the striated muscle group with cooling. However, in the striated muscle group without cooling, an increase in lesion area or perimeter lowered the quotient value. Standardised regression coefficients demonstrated that in the striated muscle with cooling, the damage zone shape was more determined by area than perimeter. However, in the parenchymal tissue, the perimeter had a more substantial impact on the damage zone shape than the area. *Conclusions*: The damage area and perimeter have predictive power on the overall shape regularity of damage zone geometry in both striated muscles and parenchymal tissue. This approach is employed to achieve a balance between the need for tumour eradication and the minimisation of ablation-induced complications to healthy tissue.

## 1. Introduction

Radiofrequency catheter ablation (RFCA) has emerged as a successful intervention method in medicine, introduced nearly three decades ago. Its approval and application have expanded across various medical fields and cases, including heart conduction system disorders [1], prostate and kidney tumours [2], and kidney denervation [3]. Moreover, RFCA has been employed to reduce arterial blood pressure [3].

Common cardiovascular disorders encompass various forms of arrhythmias stemming from different causes [4]. In 2010, approximately 33.3 million people worldwide were diagnosed with atrial fibrillation [5], with incidence increasing notably with age [6,7], exacerbating the challenge due to the ageing of society. Similarly, prostate cancer ranks as the second most commonly diagnosed cancer and the fifth leading cause of cancer death among men globally [8], with its prevalence escalating with age, peaking for men aged 70 to 80 [9]. There are no precise algorithms for the diagnosis and treatment of prostate cancer, but each new case of prostate cancer must be individually assessed and treated. Treatment should always ensure that the patient’s quality of life is not unduly compromised. Therefore, RFCA, as one of the most common non-drug therapies, is extensively utilised to treat cardiac arrhythmias [7], and it is also employed as an effective method to treat different types of cancer [8]. Although modern medicine provides a range of other cancer treatment options, including radical surgeries, external beam radiotherapy, brachytherapy, and palliative chemotherapy, the advantage of RFCA becomes particularly notable in cases where tissues are surgically unresectable. The increased utilisation of ablation as a treatment method propels the advancement of a more efficient ablation methodology.

Small tissue damage variations can be best studied through animal models, such as the dog thigh muscle tissue [10]. Dogs share similar muscle physiology, anatomy, and genes regulating muscle development, function, and repair with humans. Given their remarkable resemblance in heart connectivity and size to the human heart, canines have been used extensively in cardiovascular research [11,12]. Because of these commonalities, findings obtained using canine muscle tissues are more likely to be applicable to humans. Furthermore, transcriptomic and functional analyses have revealed that there are broad similarities in the responses to immune activation between dogs and humans in general, as well as important quantitative and qualitative differences that need to be considered when designing and evaluating cancer studies in dogs [13]. Moreover, the size and muscle mass of dogs are more similar to those of humans compared to smaller animals like mice or rats. Consequently, it is easier to perform surgical procedures and muscle biopsies that are pertinent to human clinical practice. Therefore, in experiments involving this model tissue, the thigh muscle ensures consistent contact between the ablation electrode and muscle surface throughout the RFCA procedure. However, evidence suggests that tissue histological changes often influence the electrical and thermal conductivity of the tissue, as well as heat diffusion and damage zone formation [14,15,16]. Previous studies have demonstrated that the RFCA procedure can result in the formation of elliptical lesion areas, circular lesion areas, and even more complex geometric lesions [17,18]. This phenomenon may be attributed to differences in tissue homogeneity. Therefore, in the present study, we sought to investigate whether the regularity of the shape of the lesion formed during the RFCA procedure depends on the area and perimeter.

Moreover, accurately predicting the required power for a successful and safe procedure remains challenging [19]. The amount of generated heat depends on the electrical and thermal conductivity of the tissue’s histological structures [14,15], while the extent and shape of the tissue damage area are influenced by the cooling solution [17,20]. If the parameters of RFCA are not correctly set and controlled, excess energy formed during RFCA can have a detrimental impact on adjacent tissues and organs. It is, therefore, of the utmost importance to control the ablation zone during the procedure to avoid significant damage and to protect tissue structures to the greatest extent possible. It has been demonstrated that the use of a cooling solution in RFCA can significantly mitigate tissue damage in muscular, nerve, and connective tissues [20,21]. Therefore, a 0.1% NaCl solution (C.01) was chosen for electrode cooling in this study, as it has been shown in previous studies that its application resulted in the smallest areas of damaged tissue [17]. However, despite decades of RFCA utilisation in research and medical practice, there remains a substantial demand for data analysing how this procedure depends on the different tissue conductivity properties and how to modulate the RFCA parameters (such as intensity, duration, and irrigation) to achieve optimal outcomes.

Thus, the objective of our study was to compare the changes in parenchymal tissue and striated muscle damage after RFCA with and without cooling. In addition, we aimed to evaluate the correlation between the shape regularity, area, and perimeter of the thermal lesion formed in different tissues after RFCA. This would allow us to predict the geometric changes in lesion shape.

## 2. Materials and Methods

### 2.1. Animals

The RFCA experiments were conducted on prostates and quadriceps muscles of the thigh of 13 mongrel dogs as part of a larger study in the Large Animal Clinic at the Veterinary Academy of the Lithuanian University of Health Sciences (LUHS). The experimental protocols were approved by the Bioethics Centre, LUHS, and permission for this study (approval No. 0027/2001) was granted. The dogs’ weights ranged from 10.96 to 14 (±0.05) kg. The combination of ketamine (a bolus dose of 750 mg; infusion rate 100–200 mg/h) and midazolam (a bolus dose of 25 mg; infusion rate of 3–6 mg/h) was employed for the induction of anaesthesia. The dogs were euthanised four to six hours after surgery and RFCA procedure, followed by the removal of the prostate and small pieces of quadriceps muscle tissue for histological examination in accordance with the previously outlined study protocol [18].

### 2.2. Radiofrequency Ablation Procedure

The RFCA procedure for parenchymal and muscle tissues was adapted from [17,18]. Briefly, the procedure was started by inserting an ablation catheter and the thermocouple electrode (which measured the temperature at the ablation site) into a thin pipe used for cooling via irrigation. Both electrodes were positioned perpendicular to the tissue surface. The ablation catheter tip and the thermocouple electrode were then driven to the surface of the tissue, after which a constant weight of 10 g was applied to push them in. The RFCA was conducted on each dog in two sessions, with extensive monitoring of the changes in impedance and temperature on the tissue surface. There were two RFCA sessions. In the first one, electrode cooling was not employed, whereas in the second session, a 0.1% NaCl solution (C.01) was used as the cooling irrigant (the temperature of the solution was 20 °C). This concentration was chosen because, in our previous study [17], a 0.1% NaCl solution was the most effective in reducing tissue damage. A solution of NaCl was infused at a rate of 16 mm/min for a period of 30 s during the RFCA procedure. Throughout the RFCA procedure, it was ensured that the power of 40 W, frequency of 500 kHz, and impedance ranging from 110 to 210 Ω were properly maintained. In this study, the classical 4 mm ablation catheter and a 2 mm diameter tip electrode from Biosense Webster were employed.

### 2.3. Histological Analysis

The histological tissue processing and examinations were performed at the Department of Histology and Embryology, Medical Academy, LUHS. Once collected, the samples of prostate and skeletal muscle were subjected to routine processing, embedding in paraffin, sectioning at a thickness of 2–3 μm, and staining with haematoxylin and eosin in accordance with previously outlined study protocols [22,23]. Tissue sections were selected for further analysis based on the following criteria: the absence of artefacts such as tissue tears, folds, or air bubbles and the inclusion of features necessary for analysis (necrosis zone, healthy zone, haemorrhage, and blood vessels). A total of 40 (NC) and 30 (C0.1) samples were analysed to determine the lesion area (A_Pa_) and perimeter (P_Pa_) of parenchymal tissue (Pa). In parallel, the area of damage zones (A_M_) and perimeter (P_M_) of muscular tissue (M) were calculated using 27 (NC) and 30 (C0.1) histological samples. The number of samples required for histological analysis was calculated as previously reported [24]. Images were digitised using the Olympus BX 40 microscope with an optical resolution of 2080 × 1544 pixels, where the pixel size corresponds to 3.45 × 3.45 μm. The obtained images of histological preparations were analysed and processed using the ImageJ, Microvision 1.1, and Cell Sens Dimensions 2010 software packages in accordance with the methodology employed in previous studies [17,18].

### 2.4. Mathematical Analysis

To evaluate the geometric shape of the damage zones at the ablation site, the quotient formula was employed. A quotient (Q) is defined as a ratio between the length of the principal axis and the maximum orthogonal width to the principal axis, calculated by the formula $P_{2A}=\frac{p^{2}}{4\pi A}$, where P is the perimeter and A is the area. As previously stated in [18,25], the area of a lesion is circular when the value of the coefficient is equal to 1, and the higher the value of the coefficient, the less circular the shape of the lesion.

### 2.5. Statistical Analysis

The data were analysed using the IBM SPSS Statistics 27 statistical package. The Kolmogorov–Smirnov test demonstrated that all values of the investigated characteristics exhibited a normal distribution. To compare the properties of the investigated groups, their means and standard errors were calculated. The independent samples Student’s *t*-test was employed to assess the differences between the experimental groups. The homogeneity of variances was verified by Levene’s test. Pearson’s linear correlation coefficients and regression analysis were employed to assess the statistical relationships between area, perimeter, and quotient. *p* < 0.05 was considered to indicate a statistically significant difference.

## 3. Results

### 3.1. Comparison of the Lesion Groups in Different Tissue Types

**Figure 1:**
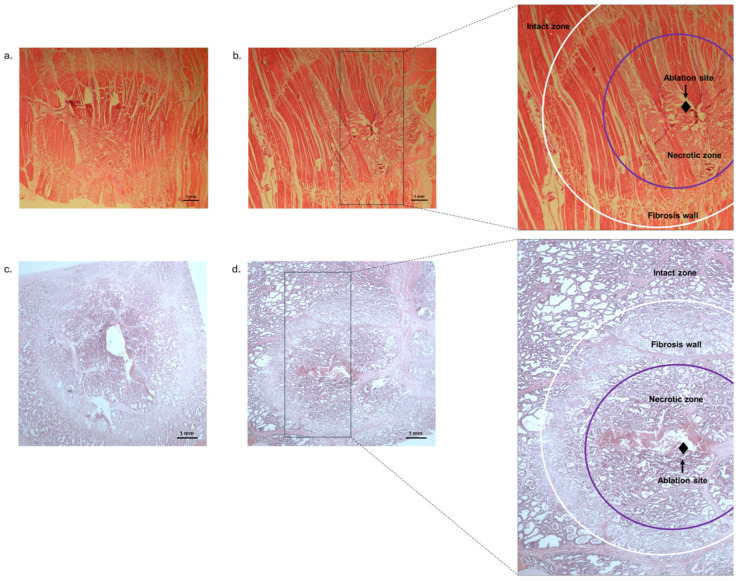
Representative haematoxylin and eosin-stained histological images of the striated muscle and parenchymal tissues after RFCA. The intact tissue is clearly distinguishable from the ablated tissue damage area, which is characterised by a coagulated necrosis zone and surrounded by a margin of fibrous tissue. (**a**): Histological image of striated muscle tissue (NC group); (**b**): histological image of striated muscle tissue (C0.1 group); (**c**): histological image of parenchymal tissue (NC group); (**d**): histological image of parenchymal tissue (C0.1 group).

We found significantly larger lesion areas in the NC group compared to the experimental session with cooling (C0.1) in the parenchymal tissue areas and in striated muscle tissue areas, as well as in the perimeters of the parenchymal tissue and in the perimeters of the striated muscle tissue (see Figure 1 and Table 1).

The experimental results also showed that the NC group samples of striated muscle tissue exhibited greater mean area, perimeter, and quotient values than those in the C0.1 group. Specifically, the NC group samples exhibited 1.5 times (*p* < 0.001) larger areas, 1.4 times (*p* < 0.001) increased perimeters, and 1.35 times (*p* < 0.001) bigger quotients (see Table 1). The quotient values were statistically significantly higher than 1 in both the C0.1 group and the NC group (*p* < 0.001).

### 3.2. Correlation between the Lesion Groups in Different Tissue Types

The correlation coefficients (see Table 2) between area and perimeter in both groups of striated muscle tissue demonstrated positive, very strong, and statistically significant correlations. In the NC group of striated muscle tissue, a moderate negative statistically significant correlation was observed between area and quotient (r = −0.438, *p* < 0.001). In contrast, no correlation was found between quotient and perimeter (r = −0.233, *p* = 0.242). We also found a moderate statistically significant positive correlation between perimeter and quotient (r = 0.515, *p* < 0.01), while no correlation was found between area and quotient (r= −0.050, *p* = 0.793) in the C0.1 group. It is worth mentioning that the damage zone shape exhibited greater irregularity in the NC groups, with the CI95% of the average quotient of the samples ranging from 1.74 to 1.84. In contrast, the irregularity of damage zone shape was significantly lower in the C0.1 groups, with the CI95% ranging from 1.28 to 1.40.

Similarly to the results obtained with striated muscle tissue, the correlation coefficients (see Table 2) demonstrated a positive, very strong, and statistically significant relationship between area and perimeter in both the NC and C0.1 parenchymal tissue groups. Moreover, positive correlations were observed between area, perimeter, and quotient were observed, with the strength of the correlation varying from medium to very strong. We noticed a strong correlation between perimeter and quotient (r = 0.933, *p* < 0.001), as well as between quotient and damage zone area (r = 0.629, *p* < 0.001) in the control group. In contrast, in the C0.1 group, the quotient correlation with damage zone perimeter (r = 0.636, *p* < 0.001) and area (r = 0.473, *p* < 0.01) was weaker than in the control group, yet still fell within the category of strong association. Furthermore, in the C0.1 group, the CI95% for the mean quotient of the samples ranged from 1.11 to 1.23, while in the group with cooling, it was significantly lower, ranging from 1.10 to 1.14.

### 3.3. The Regression Analysis in the Lesion Groups of Different Tissue Types

**Figure 2:**
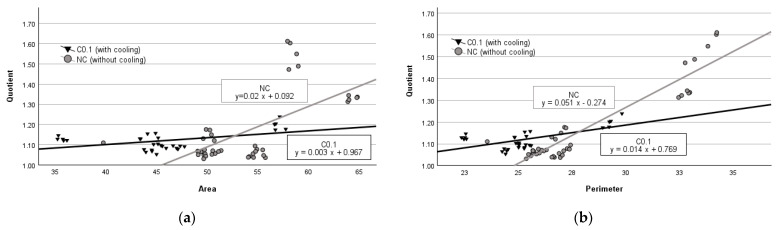
Regression analysis between area and quotient (**a**) and perimeter and quotient (**b**) in parenchymal tissue.

The regression analysis of data obtained from the group of parenchymal tissues with no cooling revealed that an average increase of 1 mm^2^ in area resulted in an average increase in quotient by 0.02 units (*p* < 0.001) (see Figure 2a), while an average increase of 1 mm in perimeter led to an average increase in quotient value by 0.051 units (*p* < 0.001) (see Figure 2b). In contrast, in the group with C.01 cooling, when the damage zone area increases by 1 mm^2^, the quotient respectively increases by 0.003 units (*p* < 0.01) (see Figure 2a), and when the perimeter increases by 1 mm it leads to an increase in the quotient value by 0.014 units (*p* < 0.001) (see Figure 2b).

**Figure 3:**
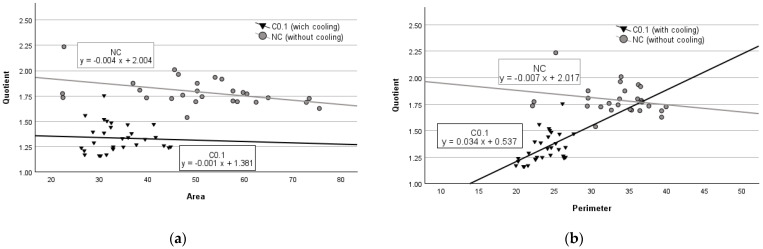
Regression analysis between area and quotient (**a**) and perimeter and quotient (**b**) in striated muscle tissue.

As expected, the results of the regression analysis of the striated muscle experiments were different from those of the parenchyma experiments. We observed that in striated muscle tissues with no cooling (NC), an increase in damage zone area by 1 mm^2^ caused the quotient value to decrease by 0.004 units (*p* < 0.001) (see Figure 3a), while a 1 mm increase in perimeter lowered the quotient value by 0.007 units (*p* = 0.242) (see Figure 3b). However, in the experimental group with cooling (C.01), an average increase of 1 mm^2^ in the tissue damage area resulted in a decrease in the quotient value by 0.001 units (*p* = 0.793) (see Figure 3a), while an average increase of 1 mm of perimeter increased the quotient by 0.034 units (*p* < 0.01) (see Figure 3b).

To determine whether the regularity of the shape of the lesion in the tissue formed during the RFCA procedure was dependent on the area and perimeter, we performed a multiple regression analysis. The results demonstrated that the regularity of the lesion shape was statistically significantly related to the area and perimeter in both parenchymal and striated muscle tissues with and without cooling (see Table 3).

The results of the multiple regression analysis indicate that in the NC and C.01 groups of both tissues, lesion shape regularity increases as the area increases (negative regression coefficients), and shape regularity decreases as the perimeter increases (positive regression coefficients). Moreover, the standardised regression coefficients revealed that only in the experimental sessions involving striated muscle tissues, when cooling was applied, the regularity of the shape of the lesion was more dependent on the area of the lesion than the perimeter. Conversely, in all other experimental groups (in both the NC and C0.1 parenchymal tissue groups and in the C.01 striated muscle tissue group) the perimeter was found to be a more significant factor in determining shape regularity than the area.

## 4. Discussion

The main findings of our study were as follows: (1) the cooling effect significantly decreased the area of injury in both striated muscle tissue and parenchymal tissue of the prostate; (2) the cooling effect caused a similar geometric shape of the damage zone at the ablation site, regardless of tissue type, and (3) the regression analysis indicated that an overall geometric shape regularity of damage zones in both types of tissue, with and without cooling, were significantly influenced by damage area and perimeter.

Literature data suggest that percutaneous radiofrequency ablation and irreversible electroporation ablation require larger ablation areas than cryoablation or microwave ablation to eradicate similar tumours [26]. In particular, ablation margins are associated with local tumour control [27]. Thus, our results showed (see Table 1) that the ablation technique had a similar effect on local tissue damage size, despite differences in the tissue types. Even in denser tissues such as striated muscles, the damaged area and perimeter were indistinguishable compared to parenchymal tissue. The electrical and thermal conductivities of the tissue in question influence the flow of current and the absorption of heat by the tissue [28], which in turn affect the ablation-caused injury. In addition, ablation technique operators may consider enlarging the size of the ablation zone [29] when using single-probe radiofrequency ablation [26] or increasing the size of the probe [30], as this is a safer method of eradicating cancer [29]. However, this is not applicable to the ablation of extremely sclerotic tumours and metastases that lack water content and associated impedance [31]. Furthermore, it is important to note that the presence of blood flow around the ablation site can reduce ablation temperature [32], thereby decreasing the effectiveness of the treatment. Moreover, there is a higher risk of local recurrence when more than one treatment session is required to treat the tumour or metastasis [33].

It has been demonstrated that an elevated temperature during the RFCA increases the risk of damage to surrounding healthy tissues or organs [26]. To achieve an optimal balance between the effectiveness of the treatment and the minimisation of damage to healthy tissue, it is essential to implement the most effective solutions by the most efficient evaluation of the treatment method. Although the tissue type can influence the outcome of the ablation procedure, our results showed that the RFCA experimental procedure with both striated muscles and parenchymal resulted in similar lesion areas and perimeters. However, irregular tumours are larger and require an additional larger area to reduce the recurrence rate. Nevertheless, there is evidence that striated muscles or parenchymal tissue without extreme sclerotisation can differently influence the ablation-caused damage size, which is one of the independent predictors of post-ablation cancer progression in different types of cancers and metastasis [29,33]. Tumour-dependent factors determining the success and survival of tumour eradication by ablation are more reliable when the tumour size is less than 3 cm [30]. The other prognostic factor is an insufficient safety margin of 1 cm on both sides of the tumour without recognisable margins or 0.5 cm with visual margins [33]. For instance, in liver cancer, tumour size, tissue scarification (cirrhosis), and location adjacent to major blood vessels [34] are predictive factors for recurrence. Notably, one of the methods for the evaluation of the ablations site is intraoperative ultrasound. This method represents the “gold” standard for imaging open radiofrequency ablations. Nevertheless, the method has been observed to overestimate the size of the ablation zone [35], particularly in instances where multiple ablations are required [30]. An alternative approach is to utilise signal-based analysis, whereby biopotentials [26], wave functions of signals [36], or post-processing techniques [37] are employed, including evaluations of outcomes. Our approach was based on the predictions of relationships between ablation-caused damage parameters and the relationship between the outcome and the size of the damaged area, perimeter, and damage zone geometry (see Table 3). The correlation coefficients (see Table 2) between areas and perimeters indicated a positive, very strong, and statistically significant correlation in both types of tissues, suggesting that a size-based relationship in the ablation sites exists, as was previously reported by Schutta and colleagues [34]. In the experimental group of striated muscle tissue without cooling (NC), a statistically significant moderate negative correlation was determined between area and quotient, while no correlation was observed between perimeter and quotient. A possible explanation for this finding is that the large sample area may have resulted in greater irregularity in the perimeter and shape of the damage zone geometry. To generate a reliable and successful treatment and to minimise complications, it is essential that the intervention can predict the extent of necrosis [30]. The estimated multiple regression coefficients indicated that the parenchymal tissue and the striated muscle tissue without (NC groups) and with the cooling (C0.1), in the event of an increased damage zone, resulted in more regular shapes (negative regression coefficients). Conversely, an increase in the damage zone perimeter resulted in the formation of more irregular shapes (positive regression coefficients).

The standardised regression coefficients indicated that in the C0.1 group of striated muscle tissue, the damage zone shape is more influenced by the area than the perimeter. In contrast, in other groups (NC_M_, NC_Pa_, C0.1_Pa_), the perimeter had a more substantial impact on the damage zone shape than the area (see Table 3). Nevertheless, since the data on ablation zone geometrical shapes were based solely on mathematical estimates, further imaging experiments would be necessary to predict tissue-dependent damage zones and their impact on the eradication of tumours or metastasis. In addition, our results demonstrated a correlation between the geometric shapes of tissue damage, perimeters, and areas in tissues that did not exhibit obvious structural changes due to RFCA. However, this approach does not permit a comprehensive understanding of the significance of shape patterns, areas, and perimeter in tumour-changed tissues and the impact of these parameters on cancer treatment. It should be also noted that other tissue characteristics, such as cellular structure density and cellular structure characteristics, were not measured. These could potentially influence the outcome of the applied ablation. Finally, the impact of the treatment on the recurrence or recovery of the treated tissue was not evaluated.

## 5. Conclusions

In conclusion, the RFCA caused similar damage areas in two different tissues, namely striated muscle tissue and parenchymal tissue of the prostate. Furthermore, cooling resulted in a reduction in the lesion area of the tissues. Additionally, regression analysis indicated that damage area and perimeter are predictive of overall shape regularity in damage zones in both striated muscles and parenchymal tissue, with and without cooling.

## Figures and Tables

**Table 1:** Values of parenchymal tissue and striated muscle areas, perimeters, and quotients after the RFCA procedure with (C0.1) and without cooling (NC).

	Parenchymal Tissue (Pa)			Striated Muscle Tissue (M)		
Parameter	NC Group (*n* = 40)	C0.1 Group (*n* = 30)	*p*	NC Group (*n* = 27)	C0.1 Group (*n* = 30)	*p*
**Area (A) (mm^2^)**	53.80 ± 0.86	45.61 ± 1.17 ^c^	<0.001	50.66 ± 2.74	33.77 ± 0.97 ^d^	<0.001
**Perimeter (P) (mm)**	28.04 ± 0.49 ^a^	25.29 ± 0.38 ^c^	<0.001	33.29 ± 0.90 ^b^	23.71 ± 0.39 ^d^	<0.001
**Quotient (Q) (unit)**	1.17 ± 0.03 ^a^	1.12 ± 0.01 ^c^	0.117	1.79 ± 0.03 ^b^	1.33 ± 0.03 ^d^	<0.001

Means marked with different letters in the row are statistically significantly different (*p* < 0.01): ^a, b^—NC group; ^c, d^—in group C0.1. The value in the p column represents the statistical significance of the difference between the NC and C0.1 groups (Student’s *t*-test for independent samples); NC—group without cooling, C0.1—group with cooling, A—area, P—perimeter, Q—quotient, Pa—parenchymal tissue, M—striated muscle tissue.

**Table 2:** Correlation coefficients of parenchymal tissue and striated muscle areas, perimeters, and quotients after RFCA procedure with (C0.1) and without cooling (NC).

	Parenchymal Tissue (Pa)				Striated Muscle Tissue (M)			
	NC Group		C0.1 Group		NC Group		C0.1 Group	
	Perimeter	Quotient	Perimeter	Quotient	Perimeter	Quotient	Perimeter	Quotient
**Area**	0.866 ***	0.629 ***	0.980 ***	0.473 **	0.966 ***	−0.438 ***	0.829 ***	−0.050
**Perimeter**		0.933 ***		0.636 ***		−0.233		0.515 **

**—*p* < 0.01; ***—*p* < 0.001 (Student’s *t*-test for independent samples). NC—group without cooling; C0.1—group with cooling; Pa—parenchymal tissue; M—striated muscle tissue.

**Table 3:** Regression analysis of damage zone area, perimeter, and quotient.

Tissue Type	Group	Attribute	Unstandardised Regression Coefficients	Standardised Regression Coefficients	*p*
Parenchymal tissue	NC	Area	−0.0228	−0.717	<0.001
		Perimeter	0.0860	1.555	<0.001
	C0.1	Area	−0.0276	−3.880	<0.001
		Perimeter	0.0971	4.440	<0.001
Striated muscle tissue	NC	Area	−0.0310	−3.234	<0.001
		Perimeter	0.0844	2.892	<0.001
	C0.1	Area	−0.0400	−1.522	<0.001
		Perimeter	0.1164	1.776	<0.001

Student’s *t*-test for independent samples. NC—group without cooling, C0.1—group with cooling.

## Data Availability

Data can be presented upon request from A.G. and G.V.
